# Functional Insertion of the Light-Induced Ion Pump KR2 into Block Copolymer Membranes

**DOI:** 10.1021/acs.biomac.6c00738

**Published:** 2026-07-16

**Authors:** Piotr Jasko, Moritz S. Muthwill, Maryame Bina, Daniel Frey, Cora-Ann Schoenenberger, Richard A. Kammerer, Cornelia G. Palivan

**Affiliations:** † Department of Chemistry, 27209 University of Basel, Mattenstrasse 22, Basel CH-4002, Switzerland; ‡ PSI Center for Life Sciences, Paul Scherrer Institute, Villigen CH-5232, Switzerland; § Swiss Nanoscience Institute, Klingelbergstrasse 82, CH-4056 Basel, Switzerland; ∥ NCCR Molecular Systems Engineering, Mattenstrasse 22, CH-4058 Basel, Switzerland

## Abstract

Light-driven membrane proteins are attractive functional elements for biohybrid membranes, as they enable direct conversion of light energy into ion gradients. While microbial rhodopsins have been extensively studied in lipid bilayers, their controlled incorporation into synthetic polymer membranes remains challenging. Here, we investigate the reconstitution of the light-driven proton and sodium pump *Krokinobacter eikastus* rhodopsin 2 (KR2) into amphiphilic block copolymer membranes using a mild detergent-assisted strategy. KR2 and a C-terminal GFP fusion variant were incorporated into both solid-supported planar membranes and polymersomes under low concentrations of *n*-dodecyl-β-d-maltopyranoside. Membrane stability and protein incorporation were characterized using surface-sensitive and fluorescence-based techniques, while ion transport activity was assessed in polymersomes. The GFP fusion enabled quantitative assessment of membrane association and insertion behavior. Reconstituted KR2 variants retained light-driven ion transport activity in polymersomes. This straightforward approach supports the development of biohybrid systems with potential for light-driven ion transport, sensing, and energy-conversion applications.

## Graphical abstract

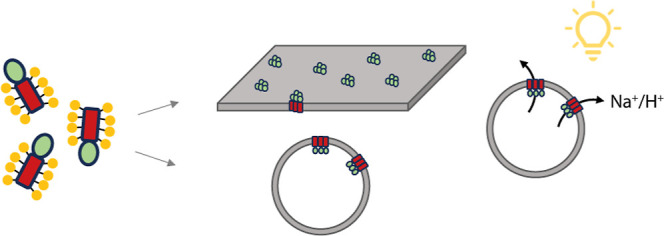

## Introduction

Membrane proteins (MPs) that convert light into ion gradients represent key functional elements for constructing biohybrid systems aimed at energy transduction and sensing.^1^ Microbial rhodopsins are a prominent class of such proteins, as they directly couple photon absorption to directional ion transport across biological membranes.^2^ In lipid-based systems, several rhodopsins, including bacteriorhodopsin (BR) and proteorhodopsin (PR), have been extensively studied and reconstituted into vesicles and planar bilayers, where they generate proton gradients across membranes that drive ATP synthesis or other energy-dependent processes in native-like assemblies.^3–6^ Extending these concepts to synthetic membranes is particularly attractive, as polymer-based systems offer enhanced mechanical robustness, chemical tunability, and long-term stability compared to lipid membranes.^7–9^ However, implementation in polymeric membranes remains substantially more challenging. While selected rhodopsins have been successfully reconstituted into polymer membranes,^6,10,11^ fundamental differences in physicochemical membrane properties, most notably altered detergent–membrane interactions, distinct polymer chain dynamics, increased membrane thickness, and constraints associated with planar and vesicular geometries, complicate MP insertion and stabilization within synthetic membranes.^6,12^ As a result, only simpler peptide-based pores have been explored in different membrane architectures,^13^ and generalizable reconstitution strategies applicable across diverse polymer membrane geometries are still missing.

Amphiphilic block copolymer membranes do not follow predictable detergent–membrane interaction rules, instead exhibit strong dependence on polymer chemistry, block flexibility, and detergent identity.^14^ Because MPs are typically handled in detergent-solubilized form, this widespread incompatibility between polymer membranes and detergents represents a fundamental barrier to efficient reconstitution. As a result, many established MP reconstitution strategies rely on partial or complete polymer solubilization prior to protein incorporation, followed by slow detergent removal by dialysis or adsorption onto Bio-Beads.^12^ These procedures are laborious and can compromise membrane integrity, as incomplete detergent removal may leave residual detergent that alters membrane permeability or mechanical stability.^15^

These challenges are further compounded by the geometry of the membrane system. Planar (two-dimensional; 2D) polymer membranes provide a continuous, curvature-free environment with uniform accessibility and are widely used for mechanistic studies, biosensing interfaces, and energy-conversion platforms.^16,17^ Vesicular (three-dimensional; 3D) polymer membranes, by contrast, present a closed and curved topology that more closely mimics cellular or organelle compartments but imposes additional geometric constraints on protein insertion and organization.^18,19^ Approaches exist in which planar proteomembranes are generated by first reconstituting MPs into polymersomes and subsequently spreading these proteopolymersomes onto solid supports.^20^ In such instances, MP incorporation occurs in the curved vesicular membrane prior to planar membrane formation, a sequence which precludes the deconvolution of curvature-dependent insertion from the structural rearrangements of the membrane assembly induced during vesicle spreading. Consequently, insertion into a truly planar polymer membrane was rarely examined in isolation from the vesicular precursor state.^21^ This limitation is further emphasized by observations that increasing membrane protein content in polymersomes can induce pronounced morphology transitions, from vesicles to stacked lamellae or even two-dimensional crystals,^15,22,23^ indicating a strong interdependence between membrane curvature and protein density. Together, these findings suggest that membrane geometry can influence protein incorporation and membrane organization. Yet, reconstitution strategies that enable protein insertion to be investigated independently in both planar and vesicular polymer membranes under comparable conditions remain limited.

In the present work, we address the above challenges by examining the incorporation of *Krokinobacter eikastus* rhodopsin 2 (KR2) into both supported polymer membranes (2D) and polymersomes (3D) using a common mild detergent-assisted approach. We selected KR2 as a model protein as it represents a compact and versatile biological module for light-driven ion transport in synthetic environments. KR2 is a seven-transmembrane α-helical retinal protein, which functions primarily as a light-driven sodium pump and switches to proton transport in the absence of sodium. Upon photoactivation, KR2 generates an outward ion flux, converting light energy into a transmembrane electrochemical gradient.^24^ Its ion selectivity can be further modulated by single-point mutations that enable transport of alternative cations such as K^+^ or Cs^+^.^25^ KR2 operates as a pentamer, and this oligomeric assembly is essential for sodium or proton pumping.^24,26,27^ KR2 relies on an all-trans retinal chromophore covalently bound within the protein core, whose photoisomerization initiates a well-defined photocycle that drives sequential charge redistribution, helix rearrangements, and unidirectional ion translocation across the membrane.^28–30^

We focus on PMOXA_10_-*b*-PDMS_25_ membranes, whose flexible PDMS domain provides sufficient chain mobility to accommodate transmembrane proteins despite the increased membrane thickness typical of synthetic systems. Polymer membranes based on flexible hydrophobic blocks have previously been shown to support functional membrane proteins,^31^ including aquaporins^15,32^ for water transport, bacterial porins^33,34^ for selective permeability, and proton-pumping PR^6^ for light-driven processes, thus demonstrating the potential of block copolymer scaffolds for biohybrid membrane engineering. Our approach is based on using low concentrations of *n*-dodecyl-β-d-maltopyranoside (DDM), a detergent widely tolerated by membrane proteins, including KR2,^29^ in order to explore whether partial membrane plasticization is sufficient to enable stable protein insertion without complete membrane solubilization or specialized detergent-removal procedures. A C-terminal GFP fusion to KR2 is employed as a fluorescent reporter to quantify membrane incorporation and assess protein orientation.

We combine surface-sensitive techniques to quantify membrane formation and protein incorporation, including quartz crystal microbalance with dissipation monitoring (QCM-D), atomic force microscopy (AFM), and confocal laser scanning microscopy (CLSM), with vesicle-based analyses such as fluorescence correlation spectroscopy (FCS), nanoparticle tracking analysis (NTA), and functional fluorescence assays. This integrated approach enables us to probe how mild DDM-assisted conditions support KR2 insertion into both supported and vesicular PMOXA_10_-*b*-PDMS_25_ membranes while preserving protein functionality and maintaining membrane integrity under controlled conditions.

Our findings indicate that controlled detergent-mediated plasticization of polymer membranes can facilitate membrane protein incorporation without bulk membrane disruption. This unified approach across synthetic membranes of different geometries supports the development of polymer-based biohybrid membranes for light-driven ion transport, sensing, and energy-conversion applications.

## Experimental Section

### Materials

The pStaby vector was obtained from Delphi Genetics Inc. , and the kanamycin-resistant pRSFDuet-1 vector was obtained from Novagen. BL21­(DE3) *Escherichia coli* cells were obtained from New England Biolabs. Unless otherwise stated, β-d-thiogalactopyranoside (IPTG), all-trans retinal, Tris, Tris–HCl, NaCl, KCl, HEPES-KOH, glycerol, imidazole, desthiobiotin, ethanol, Triton X-100, and DNase I were obtained from Sigma-Aldrich/Merck. cOmplete protease inhibitor tablets were obtained from Roche. *n*-Dodecyl-β-D-maltoside (DDM) was purchased from Anatrace. Calcein disodium salt was purchased from Fluka. Pyranine was obtained from Sigma-Aldrich/Merck, and Sodium Green was obtained from Thermo Fisher Scientific/Invitrogen. Proteinase K was purchased from New England Biolabs .

### Cloning, KR2 Variants Expression and Purification

The KR2 and KR2-GFP constructs with a C-terminal 6xHis-tag and Strep-tag, respectively, were cloned into the ampicillin pStaby vector (Delphi Genetics Inc. Charleroi, Belgium) and kanamycin resistant pRSFDuet-1 vector (Novagen), respectively. Protein expression was performed in BL21­(DE3) *E. coli* cells. The cells grew in shaking Erlenmeyer flasks with 2× Yeast Extract Tryptone (2× YT) medium at 37 °C. Expression was induced by addition of 1 mM β-d-thiogalactopyranoside (IPTG) at an optical density at 600 nm (OD_600_) of ∼0.8. Following overnight expression at 25 °C in the presence of 10 μM all-*trans* retinal, the bacterial cultures were harvested by centrifugation at 5,000*g* for 15 min. The cell pellets were disrupted with an Avestin EmulsiFlex-C3 homogenizer at 15,000 psi in lysis buffer 20 mM Tris pH 8.0, 5% glycerol, 0.5% Triton X-100, 5 μg mL^–1^ DNase I and cOmplete protease inhibitor tablets (Roche). Cell debris was removed by centrifugation at 10,000*g* for 15 min at 4 °C, and the resulting supernatant was subjected to ultracentrifugation at 150,000*g* for 1 h at 4 °C to collect the membrane fraction. The membrane pellet was resuspended with IKA T 25 Ultra-Turrax disperser in solubilization buffer that contained 50 mM Tris pH 8.0, 300 mM NaCl, cOmplete protease inhibitors, 1% (w/v) DDM (Anatrace) and stirred overnight at 4 °C. Following overnight solubilization, the suspension was subjected to a second round of ultracentrifugation to remove insoluble material. The clarified supernatant was then applied to the appropriate affinity chromatography resin depending on the affinity tag. For His-tagged protein, the supernatant was loaded onto an immobilized metal affinity chromatography (IMAC) column pre-equilibrated with IMAC buffer (50 mM Tris–HCl, pH 8.0, 150 mM NaCl, 100 mM imidazole, 0.02% (w/v) DDM). The column was washed with the same buffer to remove nonspecifically bound proteins. Bound protein was eluted using IMAC buffer supplemented with 500 mM imidazole. For Strep-tagged proteins, the supernatant was applied to a Strep-Tactin affinity column pre-equilibrated with Strep buffer (50 mM Tris–HCl, pH 8.0, 150 mM NaCl, 0.02% (w/v) DDM). After washing with equilibration buffer, the protein was eluted using Strep buffer supplemented with 2.5 mM desthiobiotin.

The protein sample was loaded onto a HiLoad Superdex 75 prep grade 16/600 column (GE Healthcare) equilibrated with SEC buffer (50 mM Tris pH 8.0, 0.05% DDM). The elution profile was monitored at 280 nm with Shimadzu UV-2401PC spectrophotometer, and the purest fractions were concentrated, flash frozen in liquid nitrogen and stored at – 80 °C until further polymersome reconstitution.

### Photoactivity Measurements of *E. coli* Overexpressing KR2 Constructs

A 100 mL volume of 2xYT liquid culture containing overexpressing KR2 or KR2-GFP BL21­(DE3) *E. coli* was prepared as described above. Cells were washed twice with 10 mL 150 mM KCl (pH 7.4) followed by centrifugation (3200*g* for 10 min at 4 °C). Immediately before the photoactivity measurements, another washing step was performed, and the concentration of the cells was adjusted to OD_600_ = 40. A volume of 800 μL was used to measure the light-driven proton translocation activity of the KR2 constructs. The activity was monitored by recording the pH in the unbuffered extracellular solution using a micro pH-electrode with an integrated temperature sensor (InLab Micro Pro, Mettler Toledo, Columbus, OH). During the measurement, the sample was stirred, and the temperature was kept constant at 18 °C using a cooling water bath. The sample was illuminated by a 2 W, warm white (3000 K) LED lamp (JANSÖ; IKEA, Delft, The Netherlands) for 8 min during four consecutive light–dark cycles. After each period of illumination, the sample was kept in the dark for 8 min to recover. To prevent background illumination, the whole setup was guarded from light. The pH and the temperature were recorded at intervals of 30 s. The pH drift was corrected by subtracting a piecewise linear function from the raw data.

### Solvent-Assisted Polymer Deposition and KR2-GFP Injection

SAPD of amphiphilic diblock copolymer PMOXA_10_-*b*-PDMS_25_ and KR2-GFP protein injection were conducted in a QCM-D flow cell on SiO_2_ QCM-D sensors (later referred to as QCM-D sensors) according to a previously described workflow.^35^ Briefly, the SAPD protocol consisted of sequential injection steps of Milli-Q water, ethanol, polymer in ethanol (0.5 mg mL^–1^), and buffer at a flow rate of 100 μL min^–1^, each step lasting 10 min. Following membrane formation, a buffer exchange step was performed, after which KR2-GFP in 0.05% (w/v) DDM was injected. A parallel experiment, run on the second QCM-D sensor, consisted of injection of 0.05% (w/v) DDM alone. Sensors were subsequently washed with buffer prior to a second injection of KR2-GFP in 0.05% (w/v) DDM or 0.05% (w/v) DDM alone at a flow rate of 8 μL min^–1^ for 16 h. Membranes were finally washed with buffer to remove reversibly associated material under the applied rinsing conditions. Prior to polymer deposition, the frequency (Δ*f*) and dissipation (Δ*D*) of the QCM-D flow cell in the aqueous phase were equilibrated until Δ*f* variations were less than 0.2 Hz over 10 min. Data analysis was performed according to a previously reported method.^35^

### Quartz Crystal Microbalance with Dissipation Monitoring

QCM-D experiments were conducted with a QSense E1 and QSense Analyzer coupled to a peristaltic pump Reglo Digital (Ismatec, Glattbrugg, Switzerland), allowing for two simultaneous measurements. Tygon MHSL tubing with two stoppers was used for the peristaltic pump and connected to PTFE tubing with (ID 0.75 mm). QSoft 401 (v. 2.8.5) software (Biolin Scientific, Göteborg, Sweden) was used to collect and record the QCM-D sensograms. During the measurement, the resonance frequencies and dissipation of the fundamental harmonic and odd-numbered overtones (3rd–13th) were recorded. The temperature was set to room temperature (25 °C). For data fitting, thickness determination, and analysis of frequency and dissipation, the software Dfind (v. 1.2.8, Biolin Scientific) was used. For consistency and clarity, data presented in the main text and figures correspond to the seventh overtone, which provided a stable and representative signal across measurements.

### Atomic Force Microscopy

Planar polymer membranes obtained with the SAPD method were analyzed using a NanoWizard 3 AFM (JPK Bruker) and the SPM control software. A commercially available reflective gold-coated DNPS-10A cantilever (nominal resonant frequency: 65 kHz, nominal spring constant: 0.35 N m^–1^) was used to record surface topography and phase micrographs, in intermittent contact (tapping) mode, with samples immersed in 50 mM Tris–HCl pH 7.4; 150 mM NaCl buffer at room temperature. Micrographs were collected at a drive frequency between 10 and 12 kHz, with a line rate of 0.4 Hz. Micrographs were subsequently analyzed using the JPK SPM Data Processing software (v. 8.1.8.).

### Confocal Laser Scanning Microscopy

CLSM measurements were performed using an LSM 880, inverted microscope ZEISS Axio Observer (Carl Zeiss, Germany) with a water immersion objective (C-Apochromate 40×/1.2 W korr FCS M27). An Ar laser was used as excitation source with excitation transmission at 488 nm set for 1%.

For supported membrane experiments, only the KR2-GFP variant was employed, as the intrinsic fluorescence provides a direct optical readout of membrane association. This enables protein–membrane interactions to be evaluated by CLSM (spatial localization) without requiring additional protein labeling. QCM-D sensors after the polymer membrane deposition and KR2-GFP application were subjected for CLSM measurements. Before performing the measurements, a small volume of the QCM-D wash buffer was placed on a cleaned microscope slide covered with the QCM-D sensor with the polymer membrane containing KR2-GFP. Measurements were performed at room temperature, and after adjusting for a sharp image, the sample was scanned randomly throughout the surface.

### Polymersome Preparation

Synthesis and characterization of amphiphilic diblock copolymer poly­(dimethylsiloxane)_25_-*block*-poly­(2-methyl-2-oxazoline)_10_ (PDMS_25_-*b*-PMOXA_10_) were described previously.^36^

Polymersomes loaded with calcein, pyranine and sodium green as well as “empty” polymersomes were prepared using the film rehydration method. Briefly, a thin film of PDMS_25_-*b*-PMOXA_10_ (10 mg mL^–1^ polymer in EtOH) was formed by rotary evaporation of the solvent (40 rpm at 40 °C, 160 mbar for 30 min; 20 mbar for another 30 min). The polymer film was rehydrated in the dark in 50 mM HEPES-KOH pH 7.0; 50 mM KCl (buffer A) containing 50 mM calcein disodium salt (Fluka) or 10 μM sodium green; 1 mM HEPES-KOH pH 7.0; 50 mM KCl (buffer B) containing 2 mM pyranine by stirring (1000 rpm) for 24 h at room temperature (RT). Following self-assembly, polymersomes were extruded (11 times) through a 200 nm Whatman Nuclepore polycarbonate membrane. Size exclusion chromatography (SEC) using ÄKTA Go system (Cytiva, Marlborough, MA) on a Superdex 200 10/300 GL column (GE Healthcare) equilibrated in either buffer A or B was performed for further purification. Polymersomes suspensions were stored at 4 °C until further use.

### Calcein-Release Assay

Calcein-loaded polymersomes were incubated with 0.05% DDM (w/v) for 16 h at 4 °C. Fluorescence was measured on a SpectraMax id3 plate reader (Molecular Devices, USA) with excitation at 495 nm and emission at 540 nm. The addition of 0.1% Triton X-100 represented 100% calcein release from the polymersomes. The percentage of fluorescence change was calculated as the ((*F* – Finitial)/Ffinal) × 100%. Experiments were performed in triplicate. The level of significance was determined using a two-way ANOVA test using GraphPad Prism version 10.4.1. (GraphPad Software, Inc.).

### Reconstitution of KR2 Constructs into Polymersomes

After the polymersome preparation, the final protein reconstitution volume was 500 μL and the polymer concentration 5 mg mL^–1^. The necessary volume of KR2 or KR2-GFP was added to give rise to (polymer to protein ratio) PPR 30, DDM concentration was adjusted to 0.05% (w/v) and the protein-detergent-polymersome suspension stirred 200 rpm for 16 h at 4 °C. After the completed incubation, the samples were extruded 11 times with a 200 nm Whatman Nuclepore polycarbonate membrane to ensure a homogeneous solution, remove any formed aggregates and purified by eluting them through a Superdex 200 10/300 GL column (GE Healthcare) using ÄKTA Go system (Cytiva, Marlborough, MA), equilibrated with either buffer A or B.

### Light Scattering

DLS experiments were performed using a Zetasizer Nano ZSP (Malvern Instruments Ltd., U.K.) at 25 °C. A laser wavelength of 633 nm and a scattering angle of 173° were used. Measurements were carried out in triplicate and each measurement consisted of ten runs with 10 s duration. The polymersome and proteopolymersome samples were typically diluted 1:10 in buffer A or B to obtain attenuation factor between 7 and 8.

Static light scattering (SLS) experiments were performed on a light scattering spectrometer (LS instruments, Switzerland) (0.5 mg mL^–1^ polymer, 25 °C, He–Ne 21 mW laser, λ = 632.8 nm, 30° to 135°). The radius of gyration (*R*_g_) was obtained from the SLS data using Guinier plots, while the hydrodynamic radius (*R*_h_) was obtained from DLS.

### Nanoparticle Tracking Analysis

NTA was performed using a NanoSight NS 300 instrument (NanoSight Ltd., U.K.) equipped with a 532 nm laser. The samples were diluted 33 000 times and applied to the viewing chamber. Three videos of 60 s were captured at room temperature for each measurement. The NTA software (version 3.4, NanoSight) was used to analyze the movement of vesicles based on tracking each particle on a frame-by-frame basis (Brownian motion) to obtain their mean and median size, together with the estimated concentration of vesicles in solution.

### Transmission Electron Microscopy

Polymersomes and proteopolymersomes (5 μL) were adsorbed on 400 mesh copper grids for 1 min, washed with water, and blotted to remove excess liquid. Specimens were negatively stained with uranyl acetate (2%) for 10 s, washed and blotted. Transmission electron microscopy micrographs were recorded on a Philips CM100 with an accelerating voltage of 80 kV.

### Fluorescence Correlation Spectroscopy

For FCS measurements, an inverted laser scanning confocal microscope (LSM 880, Carl Zeiss, Germany) with a water immersion objective (Zeiss C/Apochromat, *M* = 40, NA = 1.2) was used. An Argon laser (wavelength 488 nm) with appropriate filter (MBS 488) was used to excite Atto488. The pinhole size (34 μm, 1 AU) was adjusted before recording FCS curves of the free dye.

For FCS measurements, 20 μL of the free GFP, KR2-GFP in 0.05% (w/v) DDM or proteopolymersomes in a buffer A, were placed on a 0.15 mm thick glass coverslip mounted on the microscope stage. Fluorescence signals from free GFP, KR2-GFP in 0.05% (w/v) DDM or proteopolymersomes were measured in real time (5 s with 30 repetitions) and autocorrelation function was obtained by a QuickFit 3.0 software calculator. The experimental autocorrelation curves for the free GFP were fitted according to eq 1 with a one-component diffusion model

1 $$G\left(\tau \right)=1+\left(1+\frac{T}{1-T}e^{-\left(\frac{\tau }{\tau_{trip}}\right)}\right)\frac{1}{N}\left[\frac{1}{1+\frac{\tau }{\tau_{D}}\sqrt{1+R^{2}\frac{\tau }{\tau_{D}}}}\right]$$

where *N* represents the average number of particles in the observation volume, τ_D_ is the diffusional correlation time and *R* is the structural parameter, set to 5. *T* is the fraction of molecules in triplet state, while τ_trip_ is the triplet time. The diffusion coefficient *D* was calculated using the relation between the *x*–*y* dimension of the confocal volume (ω_xy_) and τ_D_ as in following eq 2

2 $$\tau_{D}=\frac{\omega_{xy^{2}}}{4D}$$

Two-component diffusion model, presented in eq 3 was used for fitting the experimental autocorrelation curves for the free KR2-GFP and the KR2-GFP proteopolymersomes

3 $$G\left(\tau \right)=1+\left(1+\frac{T}{1-T}e^{-\left(\frac{\tau }{\tau_{trip}}\right)}\right)\frac{1}{N}\left[\frac{f_{1}}{1+\frac{\tau }{\tau_{D1}}\sqrt{1+R^{2}\frac{t}{t_{D1}}}}\right]+\frac{1}{N}\left[\frac{f_{2}}{1+\frac{\tau }{\tau_{D2}}\sqrt{1+R^{2}\frac{t}{t_{D2}}}}\right]$$

The number of KR2-GFP (NPK) molecules per polymersome was calculated by eq 4

4 $$NPK=\frac{counts\;per\;molecule_{proteo\;polymersome}}{counts\;per\;molecule_{Free\;KR2-GFP}}$$

The hydrodynamic radius (*R*_h_) of the proteopolymersomes was calculated using Einstein–Stokes eq 5, where *D* is the diffusion coefficient, *k*_B_—Boltzmann’s constant, *T*—absolute temperature, and η—viscosity of the surrounding medium.

5 $$D=\frac{k_{B}T}{6\pi \eta R_{h}}$$

### Limited Proteolysis

Proteinase K (800 U mL^–1^; New England Biolabs, Ipswich, MA) was used to digest exposed protein moieties of reconstituted KR2-GFP. Proteinase K was added to proteopolymersomes to a final concentration of 0.05 mg mL^–1^. Samples were incubated at 37 °C for 2 h. Subsequently, samples were concentrated using Amicon Ultra Centrifugal Filter, 100 kDa MWCO. The samples were loaded on a 4–12% SDS-PAGE Mini-PROTEAN TGX Stain-Free Precast Gel (Bio-Rad) and analyzed with a Bio-Rad ChemiDoc Imaging System W1502 and the Image Lab 4.1 software (Bio-Rad, Hercules, CA) using ultraviolet transillumination and an exposure time of 20 s.

### Photoactivity Measurements of Proteopolymersomes

To detect KR2 variants’ ability to transport protons and sodium ions across the polymer membrane under illumination, we followed pyranine and sodium green fluorescence assays. The measurements were carried out in a fluorescence spectrometer (Jasco FP-8350), illuminating the sample with a 100 W xenon lamp (Intralux 4100, Volpi), utilizing a fiber guide to place the beam directly over the sample. The excitation and emission wavelengths were adjusted to 406 ± 10 nm; 460 ± 10 nm and 510 ± 10 nm for pyranine and 507 ± 10 nm and 532 ± 10 nm for sodium green by using a band-pass filter (Thorlabs). The samples were diluted twice in buffer A for sodium green assay or buffer B for pyranine assay from the corresponding vesicles SEC pool and left in the dark for 30 min for equilibration. Afterward the measurement was carried out under illumination, whereby the illumination was cycled between 50 s on and 10 s off. The fluorescence measurement was done during the off cycle to avoid interference. After the illumination measurement, the sample was measured for another 30 min in the dark to observe the re-equilibration of the fluorescence signal. The measurement data after the first 30 min in the dark was used for a correction in order to remove potential artifacts.

Passive Na^+^ permeability was assessed using sodium green-loaded polymersomes lacking KR2 or KR2-GFP. These control polymersomes were incubated in the dark with 100 mM NaCl for 30 min under the same conditions used before illumination in the sodium green transport assay. The resulting fluorescence change was normalized to the fluorescence increase obtained after complete vesicle disruption with 0.1% (v/v) Triton X-100, which was defined as the end point signal.

## Results and Discussion

### Expression, Purification, and Native Functionality of KR2 and KR2-GFP

To establish a reliable source of functional protein for subsequent reconstitution studies, wild-type KR2 and its C-terminal GFP fusion variant (KR2-GFP) were heterologously expressed in *E. coli*. A C-terminal GFP fusion was employed as a fluorescent reporter, as such constructs have previously been shown to retain fluorescence and to be compatible with membrane protein reconstitution in PMOXA_17_-*b*-PDMS_65_-*b*-PMOXA_17_ polymersomes.^6^ Plasmid constructs encoding KR2 or KR2-GFP were transformed into *E. coli* and expressed under inducible conditions (see Methods).

Protein functionality was first evaluated in transformed *E. coli* BL21 (DE3) cells using a whole-cell photoactivity assay (Figure 1A). Upon illumination of transformed cells, a characteristic decrease in extracellular pH was observed, consistent with outward proton pumping. During dark intervals, the extracellular pH gradually returned to its initial preillumination value in the surrounding unbuffered medium. When normalized for cell density, KR2-GFP exhibited a slightly larger pH shift than KR2 alone, which we attribute to partial spectral overlap between GFP emission and the all-trans-retinal absorption band, potentially enhancing local excitation efficiency.^37,38^

**Figure 1:**
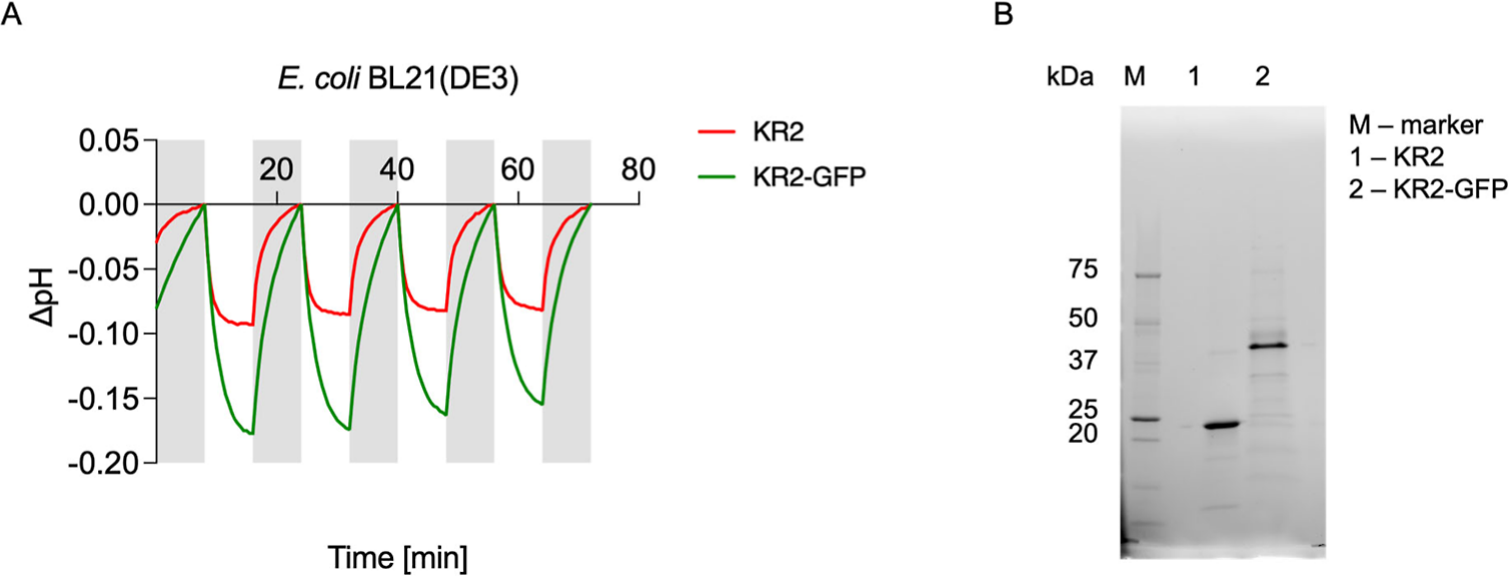
Functional and biochemical characterization of KR2 and KR2-GFP variants. (A) Light-driven extracellular pH changes measured in intact *E. coli* BL21 (DE3) cells expressing KR2 (red) or KR2-GFP (green). Gray regions indicate dark periods; white regions correspond to illumination periods. (B) SDS-PAGE analysis of purified KR2 variants. Lane M, molecular weight marker; lane 1, KR2 (∼32 kDa); lane 2, KR2-GFP (∼62 kDa).

To obtain purified KR2 for subsequent reconstitution experiments, membrane fractions were solubilized in DDM and subjected to affinity purification followed by size-exclusion chromatography (SEC). KR2 eluted as a single dominant peak corresponding to a monomeric species (Figure S1). KR2-GFP showed a main monomeric peak together with an earlier-eluting fraction, likely corresponding to higher-order species; only the monomeric KR2-GFP fraction was pooled and used for subsequent reconstitution experiments. SDS-PAGE analysis of the pooled monomer fractions (Figure 1B) revealed a single predominant band for both KR2 and KR2-GFP. In both cases, the proteins migrated slightly faster than their theoretical molecular weights, a behavior commonly observed for integral membrane proteins.^39^

### Solid-Supported PMOXA_10_-*b*-PDMS_25_ Membranes and their Interaction with KR2-GFP

To characterize the interaction of KR2-GFP with polymer membranes under well-defined conditions, we first generated solid-supported PMOXA_10_-*b*-PDMS_25_ membranes on silica surfaces using solvent-assisted polymer deposition (SAPD).^35^

Supported polymer membrane formation was monitored in real time using QCM-D (Figure 2A), which is highly sensitive to nanogram-level mass changes and provides insights into the viscoelastic properties of soft membranes.^40^ Adsorption of PMOXA_10_-*b*-PDMS_25_ onto silica produced a characteristic frequency decrease (Δ*f* = −54.8 ± 0.1 Hz), accompanied by only a small increase in dissipation (Δ*D* = 2.4 ± 0.0 ppm), consistent with the formation of a homogeneous, two-dimensional polymer membrane within the rigid regime, as expected for a solid-supported nature (Figure 2A, top panel).^35^ To visualize and characterize the surface topography of the supported membrane, we complemented QCM-D measurements by AFM (Figure S2A). The corresponding AFM height micrographs revealed a stable, laterally continuous layer with low to moderate nanoscale roughness of *R*_q_ = 2.5 ± 0.1 nm (Figure S2B). Uniform granular domains were observed, consistent with previous reports on PDMS-*b*-PMOXA and related amphiphilic block copolymer membranes prepared by solvent-exchange methods.^21,35^ Such nanoscale heterogeneity is commonly attributed to local polymer chain clustering and incomplete chain relaxation during solvent-assisted polymer deposition, where solvent exchange and evaporation kinetics govern membrane densification, roughness, and domain formation.^35,41,42^

**Figure 2:**
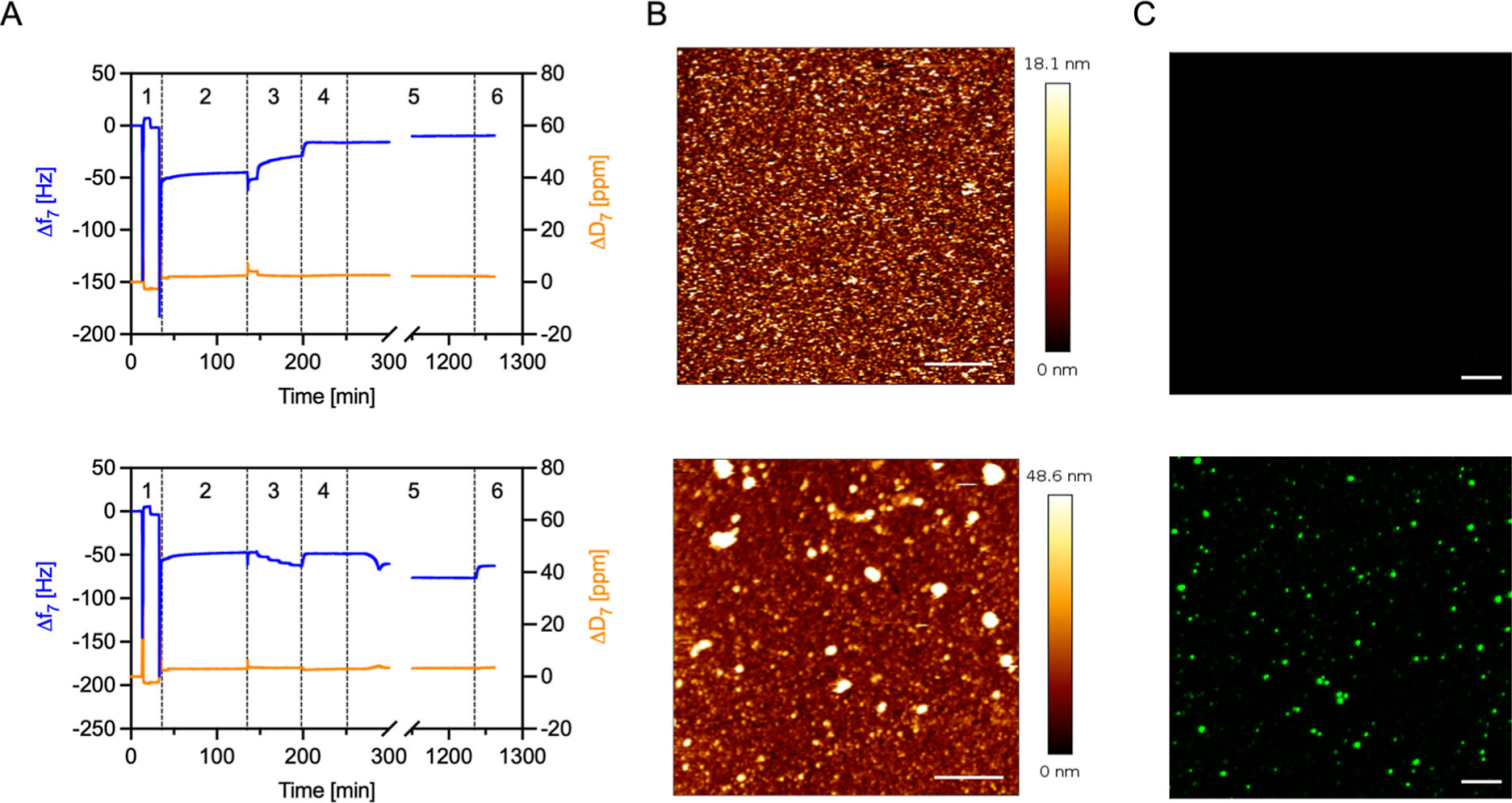
Detergent-mediated insertion of KR2-GFP into supported PMOXA-*b*-PDMS membranes monitored by quartz crystal microbalance with dissipation monitoring (QCM-D), atomic force microscopy (AFM), and confocal laser scanning microscopy (CLSM). (A) QCM-D analysis of solid-supported PMOXA_10_-*b*-PDMS_25_ membranes. Top panel: Real-time frequency (Δ*f*) and dissipation (Δ*D*) traces showing the effect of 0.05% (w/v) DDM on a preformed polymer membrane. Bottom panel: corresponding traces for KR2-GFP delivered in 0.05% (w/v) DDM. Both experiments followed the same injection sequence: (1) solvent-assisted polymer deposition (SAPD) leading to bilayer formation; (2) buffer exchange to remove unbound polymer and stabilize the baseline; (3) fast injection (100 μL min^–1^) of either DDM alone (top) or KR2-GFP in DDM (bottom). DDM alone caused an increase in Δ*f*, indicating partial mass loss from the supported polymer membrane, whereas KR2-GFP in DDM produced a net decrease in Δ*f*, consistent with protein adsorption or membrane association dominating over detergent-induced removal; (4) buffer rinse after the fast injection step. In the DDM-only experiment (control), the baseline stabilized at reduced adsorbed mass, indicating incomplete recovery after detergent exposure, whereas in the KR2-GFP/DDM experiment weakly associated material was removed and a stable baseline was re-established; (5) slow, prolonged injection (8 μL min^–1^ for 16 h) of DDM alone (top) or KR2-GFP in DDM (bottom), DDM alone led to further gradual mass loss, while KR2-GFP in DDM resulted in a continuous decrease in Δ*f*, indicative of protein association with the membrane; (6) final buffer wash. In the DDM-only case, a partially depleted membrane remained on the surface, whereas in the KR2-GFP/DDM case loosely bound material was removed while a retained protein-associated mass remained. Δ*f* and ΔD shifts are shown for the seventh overtone. (B) AFM height micrographs of supported membranes after treatment with DDM alone (top) or KR2-GFP in DDM (bottom). Scale bars: 2 μm. (C) CLSM micrographs of supported membranes treated with DDM alone (top) or KR2-GFP (bottom). Scale bars: 5 μm.

Using QCM-D, we evaluated the stability and morphology of the solid-supported PMOXA_10_-*b*-PDMS_25_ membrane by flowing buffer containing 0.05% (w/v) DDM over the membrane for 16 h (Figure 2A, top panel). This concentration provides sufficient micellar stabilization to prevent protein aggregation and allows assessment of membrane tolerance to low detergent levels prior to protein reconstitution. To enable direct comparison with protein experiments and to distinguish transient from persistent detergent effects, the same two flow regimes were applied in the absence of protein. During the initial high-flow injections (Step 3; 100 μL min^–1^ for 3 min, followed by 10 min no-flow intervals, repeated five times—Figure 2A, top panel), DDM induced an increase in frequency, Δ*f* = 15.9 ± 0.0 Hz, indicative of partial mass loss from the supported polymer membrane. Upon subsequent buffer rinsing (step 4—Figure 2A, top panel), the frequency did not return to its original baseline but increased further, Δ*f* = 12.9 ± 0.0 Hz, indicating a persistent mass loss of 64% induced by DDM and subsequent buffer injection. During prolonged low-flow exposure (Step 5; 8 μL min^–1^ for 16 h—Figure 2A, top panel), a gradual additional increase in frequency, Δ*f* = 6.6 ± 0.0 Hz, was observed, consistent with continued slow removal or thinning of polymer material. With the final buffer wash (step 6—Figure 2A, top panel), a stable plateau was achieved, corresponding to an overall mass loss of 79% relative to the baseline of the supported polymer membrane prior to detergent exposure (step 2—Figure 2A, top panel). Throughout these steps, dissipation remained largely unchanged, however the ratio of dissipation and frequency (Δ*D*/(−Δ*f*)) increased from 5.4 × 10^–8^ Hz^1–^ to 2.2 × 10^–7^ Hz^1–^, indicating a shift from rigid to more viscoelastic behavior of the remaining membrane.^43,44^ Further support for this shift was provided by AFM height micrographs (Figure 2B, top panel), which showed an increase in surface roughness in response to DDM exposure (*R*_q_ = 4.1 ± 0.3 nm; Figure S2B), compared to membrane without DDM treatment (*R*_q_ = 2.5 ± 0.1 nm). The increased roughness is consistent with detergent-induced reorganization of the polymer layer, which may reflect membrane plasticization and local restructuring rather than complete solubilization.^45^

These results indicate that SAPD-generated PMOXA_10_-*b*-PDMS_25_ planar membranes undergo partial, flow-dependent perturbation in the presence of low concentrations of DDM, resulting in a residual surface-associated polymer layer.

To evaluate KR2-GFP interaction with the supported PMOXA_10_-*b*-PDMS_25_ membrane, the protein was applied to the QCM-D chamber in the presence of 0.05% (w/v) DDM. Upon fast injection of KR2-GFP in DDM (step 3—Figure 2A, bottom panel), a decrease in frequency was observed, Δ*f* = −15.0 ± 0.1 Hz, in contrast to the mass loss seen for DDM alone, indicating that protein adsorption or membrane association dominates over detergent-induced mass removal under these conditions. To distinguish transient adsorption from stable membrane association, the same flow regimes as in the DDM control membrane were applied. In the first regime (multiple short, high-flow injections) reproducible frequency decreases during each protein pulse were observed, but the subsequent buffer wash (step 4—Figure 2A, bottom panel) restored the signal close to its baseline value of the supported polymer membrane prior to protein and detergent exposure, Δ*f* = −1.3 ± 0.0 Hz (step 2—Figure 2A, bottom panel). This behavior indicates that the initially associated KR2-GFP/DDM fraction was removed upon injection of protein- and detergent-free buffer, consistent with previous QCM-D studies in which buffer rinsing was used to remove weakly associated proteins from supported polymer membrane biointerfaces.^46,47^

In contrast, the prolonged, single low-flow injection generated a continuous frequency decrease that persisted after washing with detergent-free buffer (steps 5 and 6—Figure 2A, bottom panel). The final frequency shift of Δ*f* = −13.8 ± 0.0 Hz, compared to the step 4, corresponds to a stable mass gain by KR2-GFP of 244.9 ± 0.3 ng cm^–2^, equivalent to approximately 28% of the initially deposited membrane mass. This mass gain indicates a persistent association of KR2-GFP with the polymer membrane (step 6—Figure 2A, bottom panel). The dissipation increased by 0.6 ± 0.0 ppm upon KR2-GFP injection in slow flow, however the ratio of dissipation and frequency (Δ*D*/(−Δ*f*)) stayed in a rigid regime with a value of 5.4 × 10^–8^ Hz^1–^. This suggests that the retained protein mass is stably associated with the membrane rather than existing as a loosely attached, hydrated aggregate or detergent layer. Corresponding AFM height micrographs (Figure 2B, bottom panel) revealed an increased density of nanoscale protrusions compared to buffer- or DDM-treated control membranes. This increase in surface roughness upon KR2-GFP exposure (*R*_q_ = 9 ± 2 nm; Figure S2B) is consistent with stably membrane-associated protein. Representative AFM height profiles (Figure S2A) further showed that the increased roughness arises from localized height contributions rather than homogeneous thickening of the supported membrane. These data are therefore consistent with local KR2-GFP-associated restructuring of the supported polymer layer and formation of protein–polymer domains.

To further explore KR2-GFP integration with the membrane, CLSM imaging was employed (Figure 2C). CLSM provides a direct, spatially resolved fluorescence readout, enabling visualization of membrane-associated GFP fused to KR2. KR2-GFP-treated membranes exhibited discrete GFP-positive patches distributed across the membrane area, whereas untreated membranes showed no detectable fluorescence. This fluorescence distribution indicates a spatially heterogeneous association of KR2-GFP with the supported polymer membrane.

Taken together, QCM-D, AFM, and CLSM analyses show that KR2-GFP association with supported PMOXA_10_-*b*-PDMS_25_ membranes is time-dependent and occurs under mild detergent conditions. As the supported PMOXA_10_-*b*-PDMS_25_ layer is not covalently anchored to the silica surface, exposure to detergent alone results in partial removal and reorganization of polymer material. In contrast, the presence of KR2-GFP suppresses this mass-loss behavior and leads to net mass accumulation at the membrane, indicating that protein association alters the balance between detergent-induced removal and surface retention. This suggests that KR2-GFP contributes to stabilization of the assembly under these conditions. While QCM-D does not resolve the molecular details of this process, the combined data support the formation of a stable protein–polymer assembly.

### Reconstitution of KR2 and KR2-GFP into Polymersome Membranes

We next sought to determine whether KR2 could be reconstituted into 3D polymersome membranes and whether the protein retained its light-driven ion-pumping activity in this architecture. Prior to reconstitution of KR2 variants into polymersomes (for detailed characterization of polymersomes, see SI), we verified that the presence of DDM did not compromise PMOXA_10_-*b*-PDMS_25_ vesicle integrity, as membrane curvature in vesicles is fundamentally different from that of supported membranes. We encapsulated calcein at self-quenching concentration in polymersomes (Figure S4) to which 0.05% (w/v) DDM was added. Any membrane perturbation or dissolution would cause rapid calcein release associated with a fluorescence increase due to calcein dequenching in bulk solution. Addition of 0.05% (w/v) DDM resulted in only minor dye release from polymersomes as assessed by kinetic calcein leakage measurements normalized to the calcein end point fluorescence of Triton X-100 solubilized polymersomes (Figure S5B), while vesicle size and monodispersity remained unchanged, demonstrating that the polymersome membrane remains intact under the detergent conditions required for KR2 handling.

In contrast to the partial, flow-dependent membrane perturbation observed for supported polymer membrane in QCM-D experiment (Figure 2A), these results indicate that polymersomes maintain structural integrity under comparable detergent conditions. Together, these findings identify 0.05% DDM as a condition that enables membrane protein reconstitution while preserving overall membrane integrity in vesicular systems.

Next, purified KR2 and KR2-GFP were mixed with preformed PMOXA_10_-*b*-PDMS_25_polymersomes in the presence of 0.05% (w/v) DDM. After 16 h, the protein-detergent-polymersome mixture was extruded and purified by SEC, yielding intact polymersomes according to static and dynamic light scattering (SLS/DLS), as well as negative-stain transmission electron microscopy (TEM) analysis (Figures 3A,B and S6).

**Figure 3:**
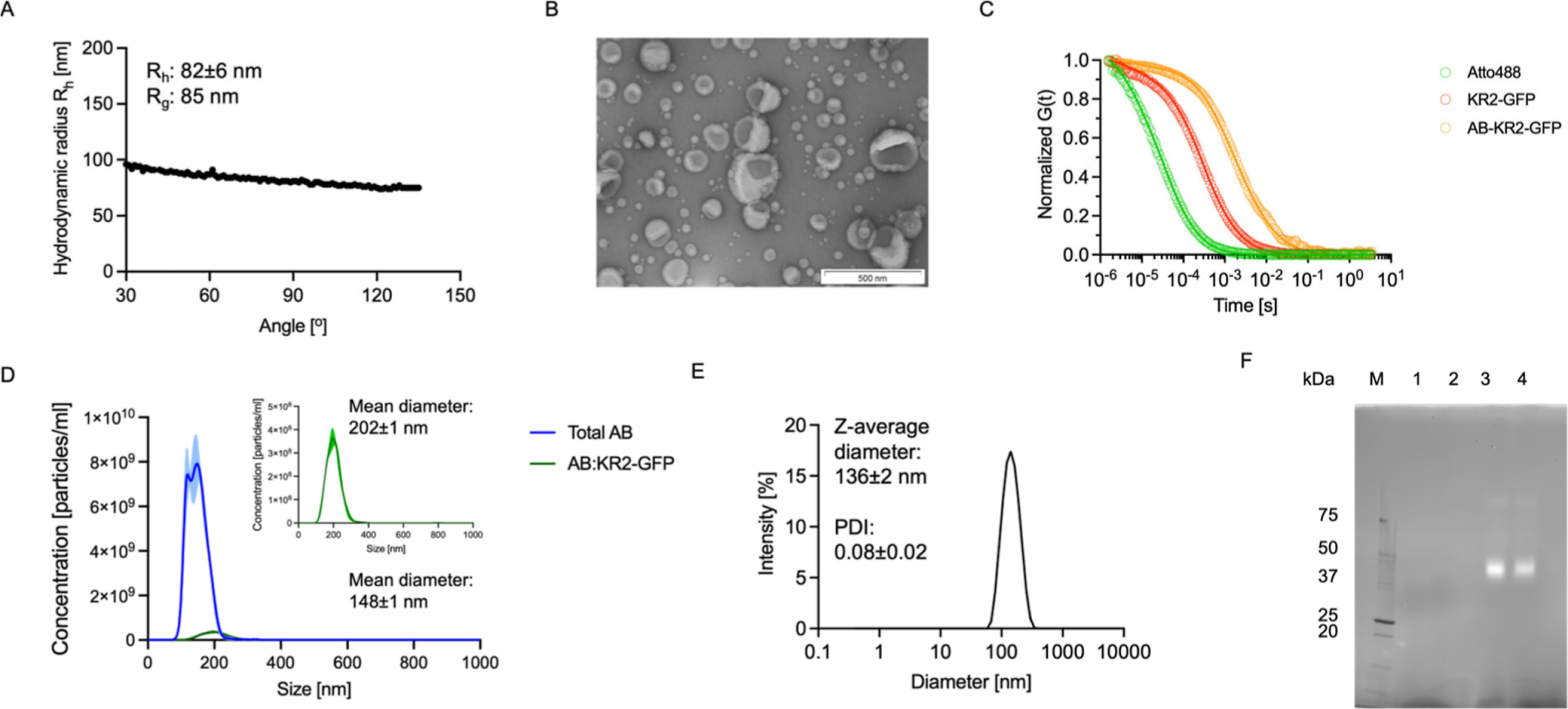
Characterization and fluorescence analysis of KR2-GFP proteopolymersomes. (A) Combined static and dynamic light scattering (SLS/DLS) analysis showing the angular dependence of the hydrodynamic radius (*R*_h_) and the derived radius of gyration (*R*_g_), confirming the presence of polymersomes as hollow spheres (*R*_g_/*R*_h_ ≈ 1). (B) Representative negative-stain transmission electron microscopy (TEM) micrograph of KR2-GFP proteopolymersomes showing the deflated morphology characteristic of intact spherical vesicular assemblies following negative staining; scale bar: 500 nm. (C) Normalized fluorescence correlation spectroscopy (FCS) autocorrelation curves of Atto488 (calibration dye), KR2-GFP in 0.05% DDM micelles, and KR2-GFP reconstituted in polymersomes. (D) Nanoparticle tracking analysis (NTA) of KR2-GFP proteopolymersomes. The blue trace represents the total vesicle population, while the green trace corresponds to the fluorescent proteopolymersome subpopulation. Inset: Representative fluorescence-mode NTA image of proteopolymersomes. Shaded regions indicate standard deviation from three independent experiments. (E) DLS analysis showing the *z*-average diameter and polydispersity index (PDI) of KR2-GFP proteopolymersomes. (F) In-gel fluorescence of proteinase K-treated KR2-GFP proteopolymersomes. Lane M, molecular-weight marker; lane 1, control polymersomes without KR2-GFP or proteinase K; lane 2, control polymersomes without KR2-GFP but treated with proteinase K; lane 3, KR2-GFP proteopolymersomes without proteinase K treatment; lane 4, KR2-GFP proteopolymersomes with proteinase K treatment.

Consistent with the approach used for supported planar membranes, the KR2-GFP fusion was employed as a protein-based fluorescent tracer, enabling visualization and quantitative analysis of KR2-GFP reconstitution in polymersomes through a combination of FCS, NTA, and in-gel fluorescence (Figure 3). FCS analysis of SEC-purified KR2-GFP proteopolymersomes showed a pronounced increase in diffusion time from 453 ± 38 μs for KR2-GFP in DDM micelles to 5795 ± 935 μs after reconstitution (Figure 3C), consistent with the presence of fluorescent vesicles. The corresponding diffusion time translated into an apparent hydrodynamic diameter of 214 ± 34 nm for the fluorescent vesicle population. Independent fluorescent NTA measurements yielded a closely matching mean diameter of 202 ± 1 nm (Figure 3C,D), confirming that both techniques probe the same population of KR2-GFP proteopolymersomes.

Beyond size determination, molecular brightness analysis of the FCS data was performed to estimate the average number of KR2-GFP molecules per fluorescent vesicle. By comparing the vesicle brightness to that of monomeric GFP references under identical optical conditions, we estimated an average of 6 ± 1 KR2-GFP molecules per fluorescent vesicle, corresponding approximately to one pentameric equivalent per fluorescent proteopolymersome. This apparent copy number is compatible with the known tendency of KR2 to form higher-order, including pentameric assemblies in membrane-like environments,^26^ although FCS does not provide structural resolution of the oligomeric state. The apparent difference between the purified monomeric state and the membrane-associated oligomeric state is therefore not unexpected. Detergent-solubilized purification can favor monomeric species, whereas lateral confinement, hydrophobic matching, local protein concentration, and protein–protein interactions within the membrane can promote reassembly into higher-order states.

To assess the entire vesicle population, DLS measurements were performed after SEC purification, revealing a monomodal size distribution with a mean diameter of 136 ± 2 nm (Figure 3E). Total NTA analysis yielded a mean diameter of 148 ± 1 nm (Figure 3D), slightly larger than the DLS value, which is consistent with methodological differences between intensity-weighted DLS and number-weighted NTA measurements. In contrast to total DLS and NTA measurements, FCS and fluorescent NTA selectively detected only the KR2-GFP proteopolymersomes, revealing a larger mean diameter for this fluorescent vesicle population. Comparison of total and fluorescent NTA particle counts indicated that approximately 5% of the total vesicle population exhibited detectable fluorescence. The preferential detection of KR2-GFP in larger vesicles suggests that protein incorporation may not occur uniformly across all polymersomes and could depend on vesicle size.

In addition to serving as a qualitative fluorescent reporter for membrane reconstitution, the GFP fusion tag also provides a topological marker for KR2 insertion. Fluorescent protein tags have been used in synthetic membrane systems to direct and report on the membrane-protein orientation.^200^ In our system, the C-terminal GFP thus enables both the optical identification of KR2 proteopolymersomes and the assessment of protein orientation once inside the synthetic membrane of polymersomes. To determine protein orientation, we performed a qualitative protease-accessibility assay by limited proteolysis of SEC-purified KR2-GFP proteopolymersomes with proteinase K, followed by in-gel fluorescence analysis (Figure 3F). Fluorescent bands corresponding to KR2-GFP were detected both before and after proteinase K treatment, whereas control polymersomes lacking protein showed no fluorescence signal. The persistence of the GFP fluorescence after digestion demonstrates protection of the C-terminal GFP domain, consistent with a luminal orientation of the KR2 C-terminus. In addition, as DLS/NTA and negative-stain TEM showed proteopolymersome populations without evidence of pronounced aggregation, it indicates that the retained GFP fluorescence primarily reflects membrane-protected KR2-GFP rather than externally adsorbed protein aggregates.

For untagged KR2 proteopolymersomes, SEC-purified samples were analyzed by SDS-PAGE, where a single major protein band was observed, while control polymersomes without protein showed no detectable bands (Figure S6B). DLS measurements of KR2 proteovesicles yielded size distributions indistinguishable from those of KR2-GFP samples (136 ± 2 nm; Figure S6A), indicating that the GFP tag does not measurably affect vesicle size or structural integrity.

Together, these results demonstrate that PMOXA_10_-*b*-PDMS_25_ polymersomes can accommodate both KR2 and KR2-GFP under mild detergent conditions without detectable disruption of the vesicular architecture (Figures 3 and S6A). The use of SEC as a postreconstitution cleanup and vesicle fractionation step, without the need for dialysis or Bio-Beads, provides a simple and reproducible procedure for membrane protein reconstitution into polymer vesicles.

### Light-Driven Ion Transport by Reconstituted KR2 and KR2-GFP

To assess the functional activity of reconstituted KR2 variants, light-driven ion transport across polymersome membranes was monitored using fluorescence-based assays with the encapsulated reporters pyranine and sodium green (SG) (Figures S7 and S8). Pyranine is a ratiometric pH-sensitive fluorophore whose fluorescence intensity ratio between excitation at 460 and 406 nm (I460/I406, emission at 510 nm) decreases upon acidification of the vesicle lumen and increases upon alkalization.^6,48^ On the other hand, SG is a fluorescent indicator whose emission intensity increases with rising sodium concentration. Both dyes were encapsulated during polymersome formation by inclusion in the rehydration buffer used for vesicle self-assembly, followed by SEC purification of resulting polymersomes to remove nonencapsulated dye (see methods).

Upon illumination, KR2 proteopolymersomes exhibited a clear decrease in the pyranine fluorescence ratio (Figure 4A). This negative shift reflects lumen acidification, consistent with light-driven proton transport to the polymersome interior. When illumination was stopped, the ratio gradually returned toward its initial value, indicating that the light-induced signal change was reversible under the applied conditions.

**Figure 4:**
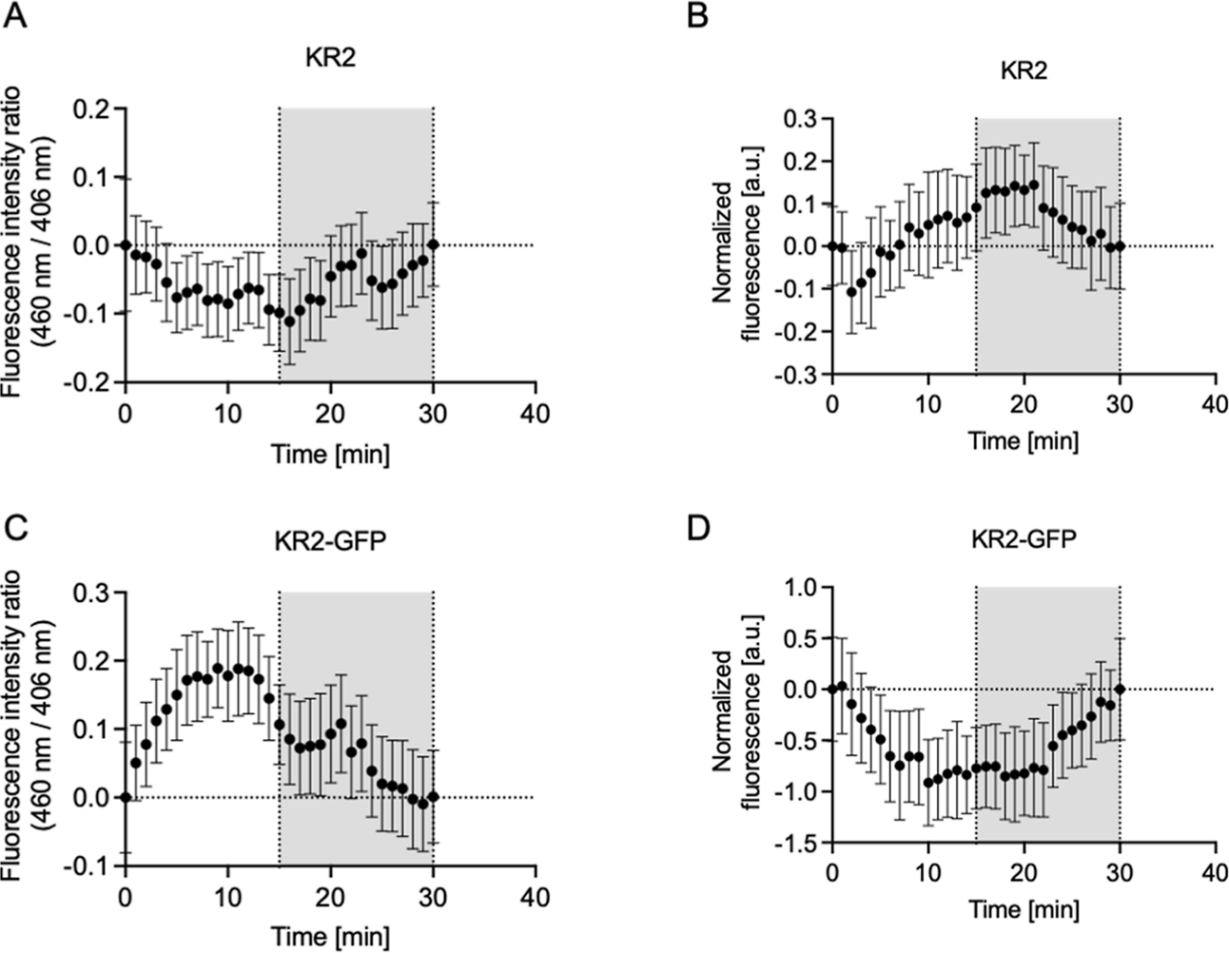
Light-driven ion transport activity of KR2 and KR2-GFP proteopolymersomes. (A) Pyranine-based pH assay of proteopolymersomes. White regions indicate illumination periods; gray regions correspond to dark intervals. A negative shift in the fluorescence ratio denotes lumen acidification consistent with proton pumping. (B) Sodium green (SG) assay of KR2 proteopolymersomes in the presence of 100 mM external NaCl, consistent with light-dependent modulation of intravesicular Na^+^ concentration. (C) Pyranine measurements of KR2-GFP proteopolymersomes display an opposite fluorescence response compared to KR2, suggesting an inverted protein topology in which the C-terminal GFP faces the vesicle lumen. (D) Sodium green fluorescence measurements of KR2-GFP proteopolymersomes suggest a light-dependent reduction in intravesicular Na^+^ concentration, consistent with Na^+^ export from the vesicle lumen. For each experiment, fluorescence traces from control polymersomes lacking protein were subtracted from the corresponding proteopolymersome traces to correct for background signal. Data represent the mean of three independent background-corrected experiments (*n* = 3), with error bars calculated by propagation of the standard deviations from both control and proteopolymersome measurements.

To probe KR2 sodium pumping activity, polymersomes were incubated with 100 mM external NaCl prior to illumination. Since passive Na^+^ equilibration could influence the interpretation of the SG response, we evaluated Na^+^ permeability in control polymersomes lacking KR2 or KR2-GFP under the same preillumination conditions. Only a negligible fluorescence increase was detected, amounting to 2.7 ± 0.3% of the maximum fluorescence measured after Triton X-100 (Triton X-100 end point signal; Figure S9). This indicates that PMOXA_10_-*b*-PDMS_25_ polymersomes exhibit very low passive Na^+^ permeability on the time scale of the experiment. Thus, passive Na^+^ entry during the preincubation step is limited and cannot account for the light-dependent SG response observed in KR2-containing proteopolymersomes. Upon illumination of KR2 proteopolymersomes, a change in SG fluorescence was observed (Figure 4B), indicating light-dependent modulation of the intravesicular sodium concentration. The relatively modest signal amplitudes observed in both pyranine and SG assays likely reflect a mixed population of KR2 orientations within the membrane. Nevertheless, the direction of the fluorescence responses indicates that a larger fraction of KR2 molecules is oriented such that proton and sodium transport occurs into the polymersome lumen, whereas oppositely oriented proteins do not contribute to net ion transport.

KR2-GFP proteopolymersomes on the other hand, exhibited a more pronounced and inverted light-dependent fluorescence response in both functional assays (Figure 4C,D). In the pyranine assay, illumination resulted in an increase in the I460/I406 ratio, corresponding to lumen alkalization, while in the SG assay, illumination produced a decrease in fluorescence intensity, indicating a reduction in intravesicular sodium concentration. These inverted responses relative to untagged KR2 are consistent with an opposite net direction of ion transport and indicate a predominant insertion orientation in which the C-terminal GFP domain faces the polymersome lumen. This interpretation is supported by limited proteolysis experiments combined with in-gel fluorescence analysis (Figure 3F), which demonstrated proteolytic protection of the GFP moiety, consistent with a luminal localization of the C-terminus. In both pyranine and SG assays, the larger signal amplitudes observed for KR2-GFP polymersomes further suggest a more uniform protein orientation compared to untagged KR2.

Because the fluorescent dyes were encapsulated within the polymersome lumen, the observed fluorescence changes report changes in intraluminal ion concentration. In the SG assay, passive Na^+^ permeability of control polymersomes was low under the preillumination conditions (Figure S9), indicating that passive equilibration alone cannot account for the light-dependent fluorescence response. The decrease in sodium green fluorescence observed for KR2-GFP proteopolymersomes upon illumination is therefore consistent with KR2-mediated Na^+^ export from the vesicle lumen. The gradual return of the fluorescence signal toward baseline during dark periods further supports reversible ion redistribution across the polymer membrane.

Taken together, the directionality, reversibility, and light dependence of the fluorescence traces demonstrate that both KR2 and KR2-GFP are functionally reconstituted in PMOXA_10_-*b*-PDMS_25_ polymersomes. These results confirm that the polymer membrane environment supports the photoactive conformational cycle of KR2 required for light-driven proton and sodium transport.

## Conclusions

This study presents a mild and straightforward strategy for integrating the light-driven sodium pump KR2 into both two-dimensional supported polymer membranes and three-dimensional polymersomes assembled from PMOXA_10_-*b*-PDMS_25_. Using low concentrations of DDM, KR2 and KR2-GFP were successfully associated with supported polymer membranes and functionally reconstituted into polymersomes while preserving vesicle integrity.

For planar, solid-supported membranes, exposure to KR2-GFP solubilized in DDM enabled stable protein association under conditions where the polymer membrane undergoes partial, flow-dependent perturbation when treated with DDM alone. In polymersomes, the same principle allowed incorporation of both KR2 variants while maintaining vesicle integrity and supporting light-driven ion transport, as verified by fluorescence-based functional assays. These observations indicate that controlled detergent-mediated plasticization of the polymer membrane is sufficient to facilitate membrane protein insertion without inducing bulk membrane solubilization.

Importantly, the reconstitution process proceeds under mild conditions without dialysis or Bio-Beads, relying instead on buffer washing for supported membranes or SEC for vesicles. The applicability of the same detergent-assisted insertion principle across both planar and vesicular polymer membranes provides a practical route for incorporating functional membrane proteins into synthetic polymer-based membrane systems. These findings highlight how controlled detergent–polymer interactions can be used to enable membrane protein integration in polymer membranes, supporting the development of polymer-based biohybrid platforms for sensing, catalysis, and light-driven energy conversion.

## Supplementary Material



## Notes

Size-exclusion chromatography of KR2 and KR2-GFP; AFM characterization of supported PMOXA_10_-*b*-PDMS_25_ membranes; physicochemical characterization of polymersomes by DLS, multiangle SLS/DLS, NTA, and TEM; characterization of calcein-loaded polymersomes and detergent stability (size distributions and leakage assays); comparative analysis of control and KR2/KR2-GFP proteopolymersomes (DLS, SLS/DLS, NTA, SDS-PAGE, TEM); characterization of pyranine- and sodium green-loaded polymersomes and corresponding proteopolymersomes (PDF)
