# Supplementary material for: Functional Insertion of the Light-Induced Ion Pump KR2 into Block Copolymer Membranes
Source: Biomacromolecules. 2026 Jul 16;27(8):5449–60. doi: 10.1021/acs.biomac.6c00738 (PMC13463550; doi:10.1021/acs.biomac.6c00738)
Supplement: Supplementary file 1 [file bm6c00738_si_001.pdf]

Supplementary Information

Functional Insertion of the Light-Induced Ion Pump KR2 into Block-Copolymer Membranes

Piotr Jasko [1,2,3], Moritz S. Muthwill [1,4], Maryame Bina [1], Daniel Frey [2], Cora-Ann Schoenenberger [1], Richard A. Kammerer [2] and Cornelia G. Palivan [1,3,4]

[1] Department of Chemistry, University of Basel, Mattenstrasse 22, CH-4002 Basel, Switzerland

[2] PSI Center for Life Sciences, Paul Scherrer Institute, CH-5232 Villigen, Switzerland

[3] Swiss Nanoscience Institute, Klingelbergstrasse 82, CH-4056 Basel, Switzerland

[4] NCCR Molecular Systems Engineering, Mattenstrasse 22, CH-4058, Basel, Switzerland

[*] Corresponding Authors:

Richard A. Kammerer, PSI Center for Life Sciences, Paul Scherrer Institute, CH-5232 Villigen PSI, Switzerland. E-mail: richard.kammerer@psi.ch

Cornelia G. Palivan, Department of Chemistry, University of Basel, Mattenstrasse 22, CH-4002 Basel, Switzerland. E-mail: cornelia.palivan@unibas.ch

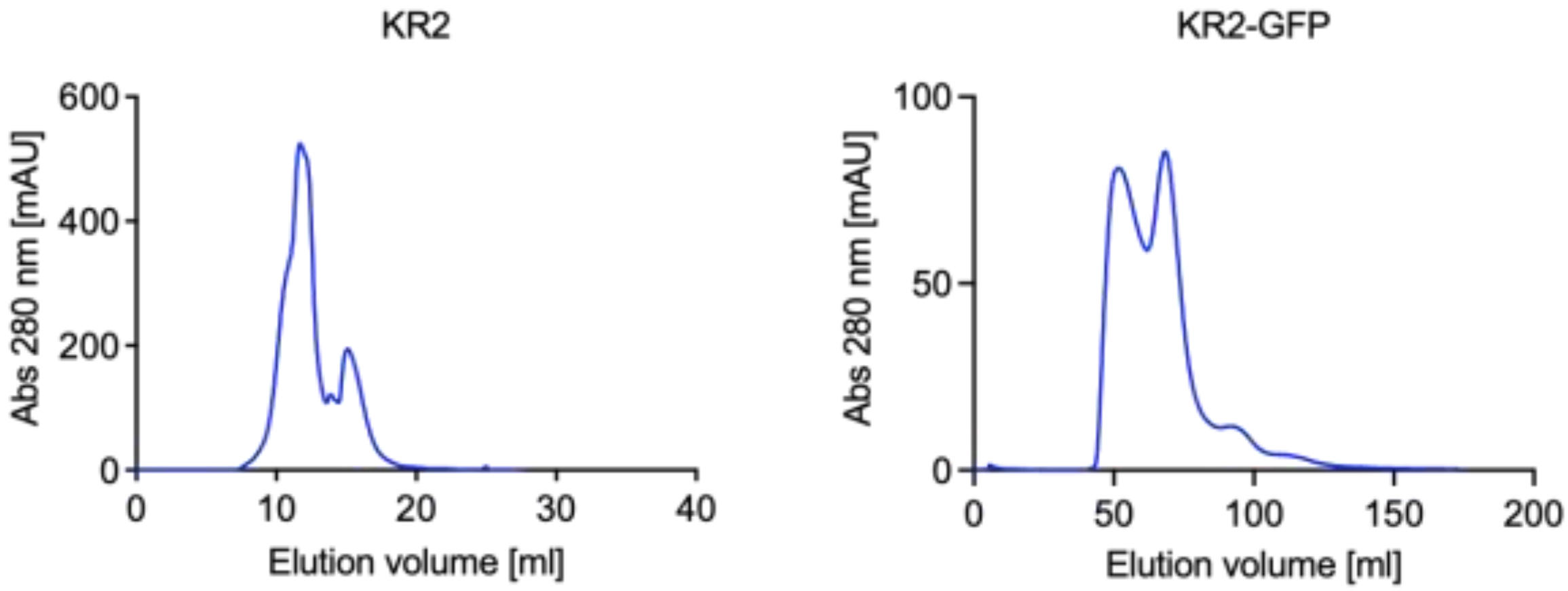

Supplementary Fig. S1. Size-exclusion chromatography (SEC) of KR2 (left panel) and KR2-GFP (right panel).

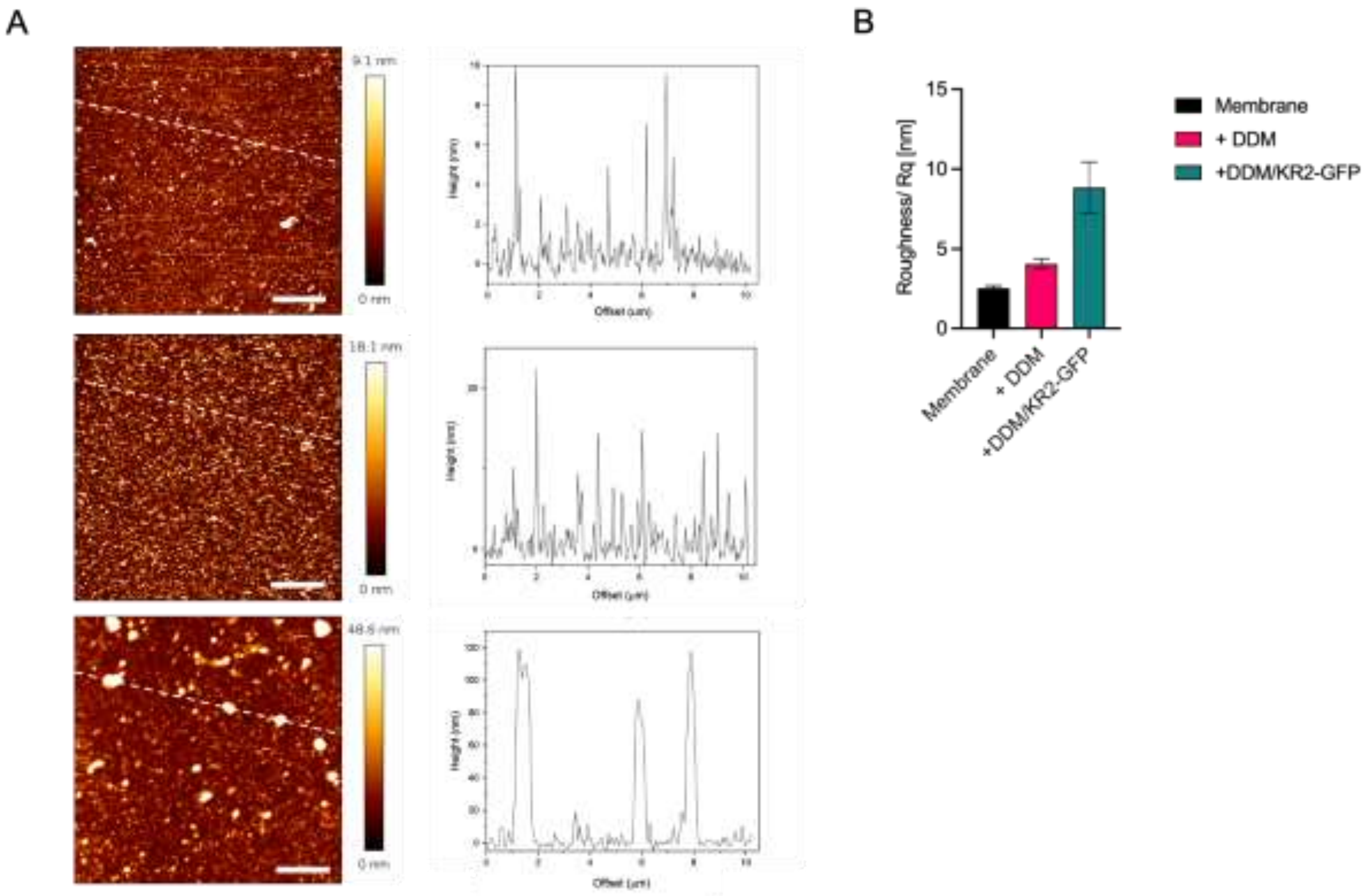

Supplementary Fig. S2. AFM analysis of supported PMOXA$_{10}$-*b*-PDMS$_{25}$ membranes. (A) AFM height micrographs and corresponding height cross-sections of supported polymer membranes. Top panel: untreated PMOXA$_{10}$-*b*-PDMS$_{25}$ membrane in buffer; middle panel: membrane after exposure to 0.05% DDM; bottom panel: membrane after exposure to KR2-GFP in 0.05% DDM. Dashed white lines indicate the regions used for height profile extraction. Scale bars: 2 µm. (B) Quantification of membrane surface roughness (Rq) for untreated membranes, DDM-treated membranes, and membranes exposed to DDM + KR2-GFP. Bars represent mean Rq values ± SD from three independent AFM images per condition.

The PMOXA$_{10}$-*b*-PDMS$_{25}$ block copolymer self-assembled into uniform vesicles under standard film-rehydration conditions (for details see Methods), yielding stable dispersions. Size and morphological characterization was performed using a combination of dynamic and static light scattering (DLS/SLS), nanoparticle tracking analysis (NTA), and transmission electron microscopy (TEM). DLS revealed a monomodal distribution with a Z-average diameter of 164 ± 2 nm (PDI 0.05 ± 0.01), in agreement with the mean diameter of 157.3 ± 0.5 nm obtained by NTA. The corresponding radius of gyration determined by SLS (R_h=99±5; R_g=103 nm) resulted in a ρ-factor (R_g/R_h) of ~1.0, characteristic of hollow spheres. TEM micrographs confirmed the spherical morphology and absence of aggregation, showing vesicles with well-defined membranes and diameters consistent with light-scattering measurements. Together, these data shows that the PMOXA$_{10}$-*b*-PDMS$_{25}$ copolymer reproducibly forms stable polymersomes suitable for subsequent reconstitution studies.

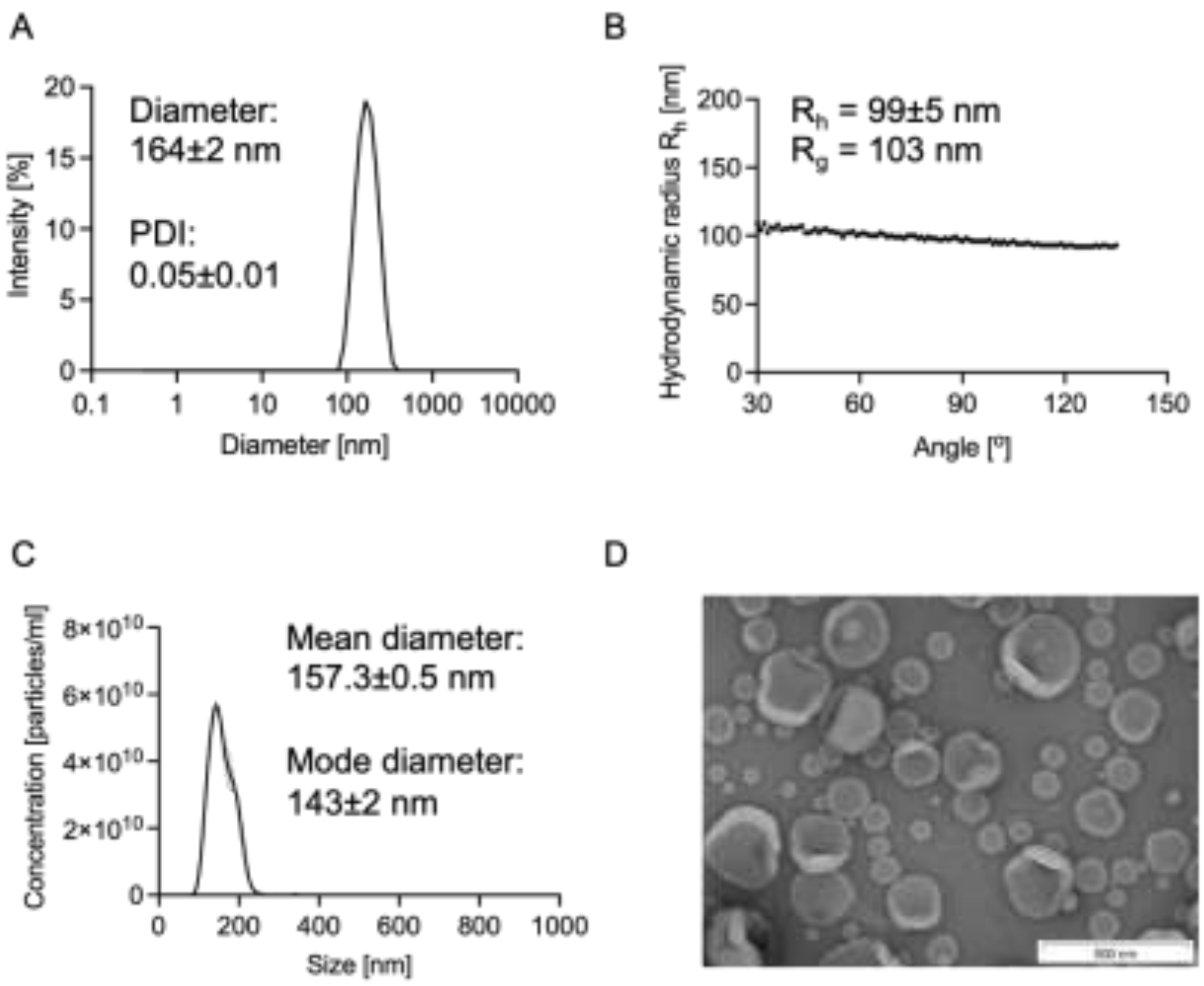

Supplementary Fig. S3. (A) Dynamic light scattering (DLS) analysis showing the *z*-average hydrodynamic diameter and polydispersity index (PDI). Measurements were performed in triplicate. (B) Combined static and dynamic light scattering (SLS/DLS) analysis showing the angular dependence of the hydrodynamic radius (R_h) and the derived radius of gyration (Rg), confirming presence of hollow spheres (Rg/Rh ≈ 1). Differences between DLS-derived diameters and R_h values reflect the distinct weighting and angular dependence of the applied light scattering techniques. (C) Nanoparticle tracking analysis (NTA) of polymersomes, showing the particle size distribution; shaded regions represent standard deviation from three independent experiments. (D) Representative negative-stain TEM micrograph displaying the characteristic spherical morphology of intact polymersomes. Scale bar: 500 nm.

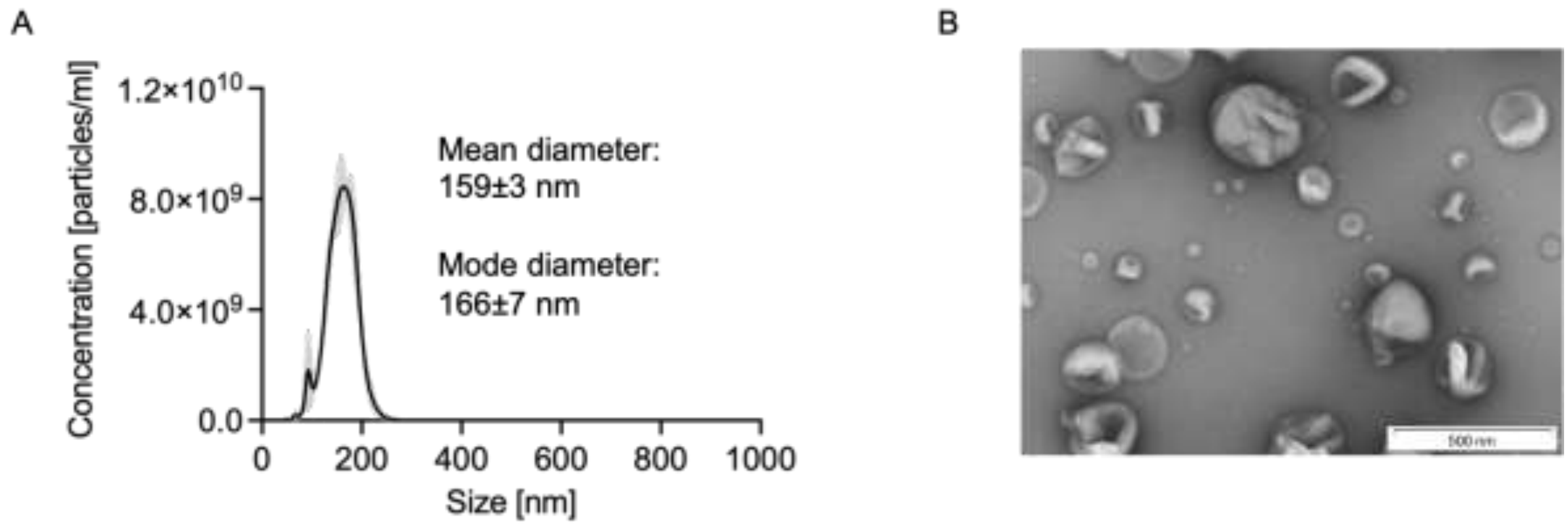

Supplementary Fig. S4. (A) Nanoparticle tracking analysis (NTA) of polymersomes loaded with calcein, showing the particle size distribution; shaded regions represent standard deviation from three independent experiments. (B) Negative stain transmission electron microscopy (TEM) of corresponding calcein-loaded polymersomes. Scale bar: 500 nm.

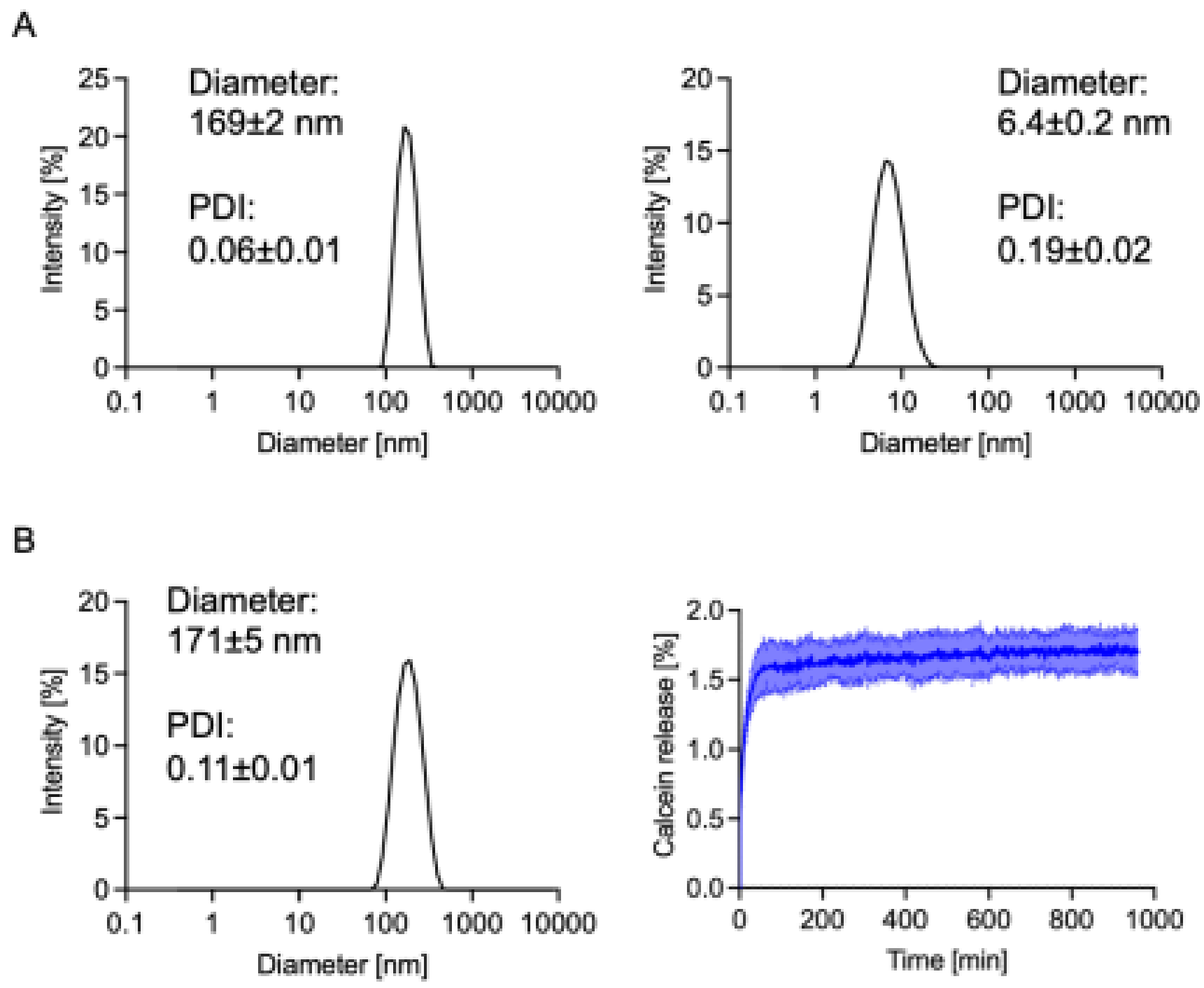

Supplementary Fig. S5. (A) Dynamic light scattering (DLS) analysis showing the *z*-average hydrodynamic diameter and polydispersity index (PDI) of calcein-loaded polymersomes (left), 0.05% DDM micelles (right). Measurements were performed in triplicate. (B) DLS profile of calcein-loaded polymersomes after incubation with 0.05% DDM showing negligible size change for DDM. The right panel shows the corresponding calcein leakage fluorescence kinetics recorded under identical conditions. Data represent the mean of three independent experiments; shaded regions indicate standard deviation.

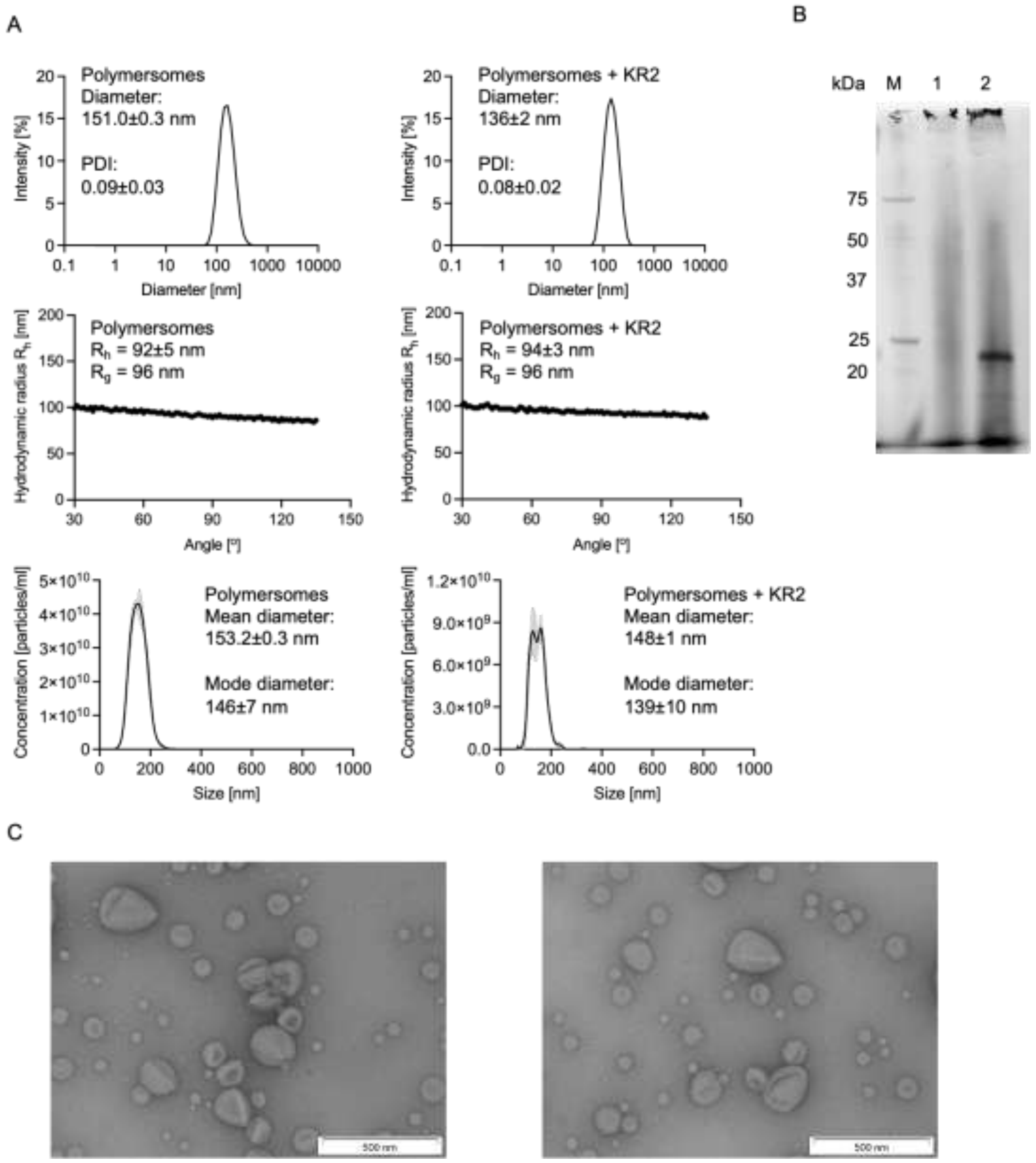

Supplementary Fig. S6. (A) Top panels: Dynamic light scattering (DLS) size distributions (z-average hydrodynamic diameter). Middle panels: Combined static and dynamic light scattering (SLS/DLS) analysis showing the angular dependence of the hydrodynamic radius (Rh) and the corresponding radius of gyration (Rg), consistent with hollow spherical vesicles (Rg/Rh ≈ 1). Bottom panels: Nanoparticle tracking analysis (NTA) size distributions. Left panels correspond to control polymersomes; right panels correspond to KR2 proteopolymersomes. Mean hydrodynamic diameters are indicated. Differences between DLS-derived diameters and R_h values reflect the distinct weighting and angular dependence of the applied light scattering techniques. DLS data represent the mean of n = 3 independent measurements. NTA distributions show the mean with shaded regions indicating the standard deviation from three replicates. (B) SDS-PAGE analysis of polymersome fractions: lane 1, control polymersomes without KR2; lane 2, KR2 proteopolymersomes; M, molecular-weight marker. (C) Negative-stain transmission electron microscopy (TEM) micrograph of control vesicles without protein (left), and KR2 proteopolymersomes (right), displaying typical spherical morphology. Scale bar: 500 nm.

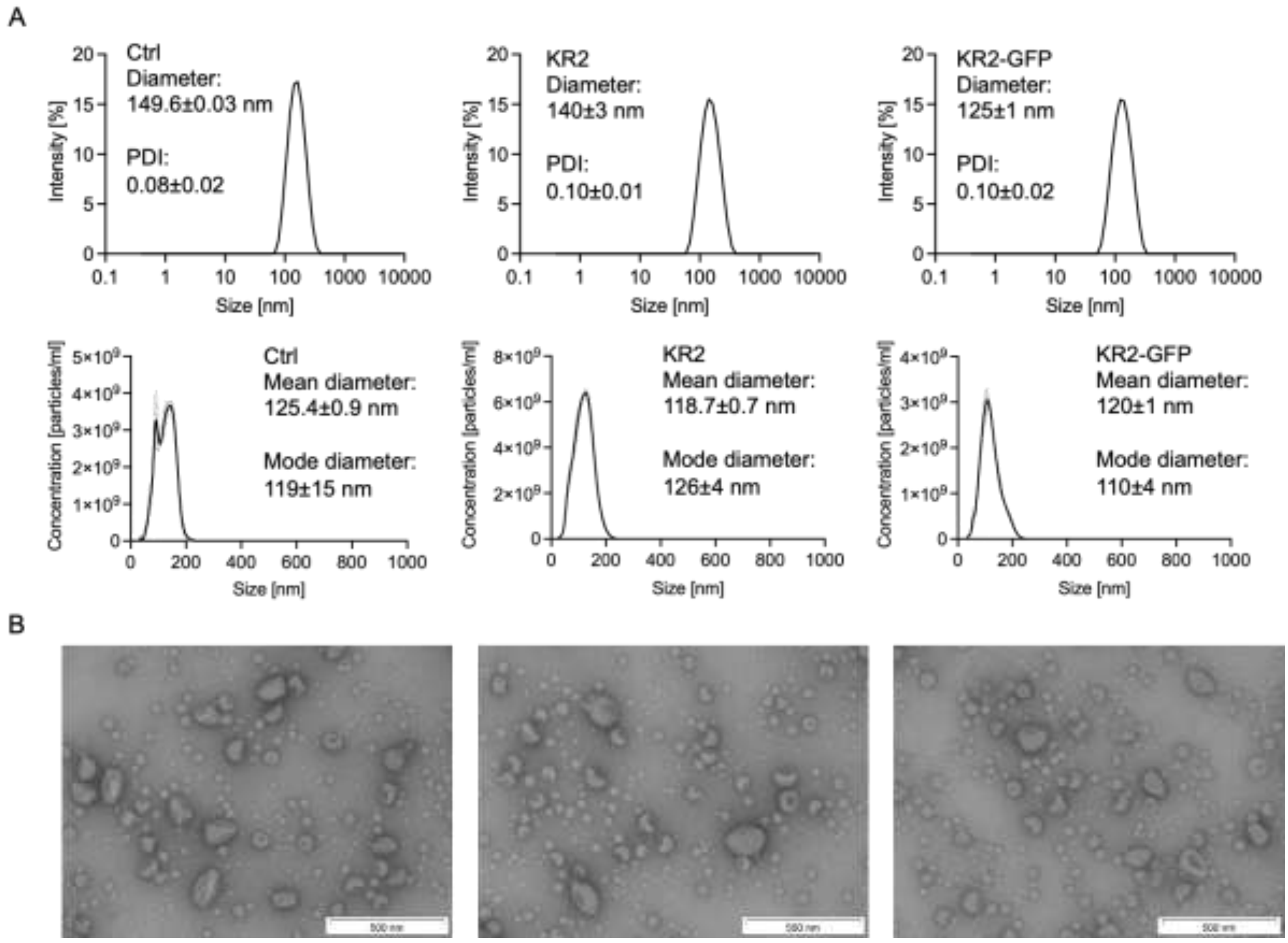

Supplementary Fig. S7. (A) Dynamic light scattering (DLS) (top panel) and corresponding nanoparticle tracking analysis (NTA) (bottom panel) size distributions of polymersomes loaded with pyranine. Control polymersomes (left); KR2 proteopolymersomes (middle) and KR2-GFP proteopolymersomes (right). Mean hydrodynamic diameters are indicated. DLS data represent the mean from n = 3 independent measurements; NTA plots show the mean distribution, with shaded regions denoting the SD from three replicates. (B) Negative-stain transmission electron microscopy (TEM) micrograph of control vesicles loaded with pyranine without protein (left); KR2 proteopolymersomes (middle); KR2-GFP proteopolymersomes (right) displaying typical spherical morphology. Scale bar: 500 nm.

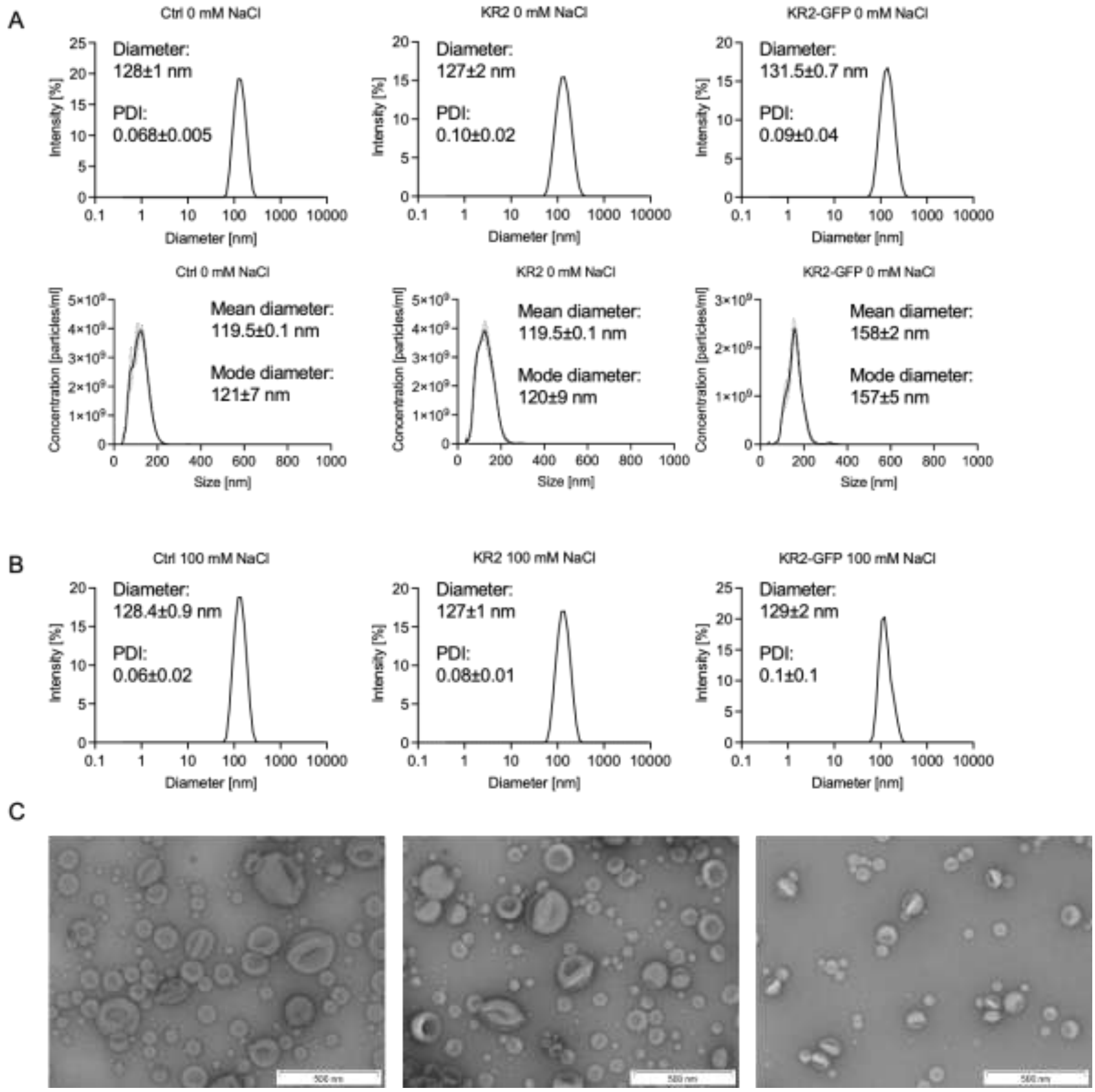

Supplementary Fig. S8. (A) Dynamic light scattering (DLS) (top panel) and corresponding nanoparticle tracking analysis (NTA) (bottom panel) size distributions of polymersomes loaded with sodium green. Control polymersomes (left); KR2 proteopolymersomes (middle) and KR2-GFP proteopolymersomes (right). Mean hydrodynamic diameters are indicated. DLS data represent the mean from n = 3 independent measurements; NTA plots show the mean distribution, with shaded regions denoting the SD from three replicates. (B) DLS size distributions of polymersomes loaded with sodium green in the presence of 100 mM NaCl. Control polymersomes (left); KR2 proteopolymersomes (middle) and KR2-GFP proteopolymersomes (right). Mean hydrodynamic diameters are indicated. DLS data represent the mean from n = 3 independent measurements. (C) Negative-stain transmission electron microscopy (TEM) micrograph of control vesicles loaded with sodium green without protein (left); KR2 proteopolymersomes (middle); KR2-GFP proteopolymersomes (right) displaying typical spherical morphology. Scale bar: 500 nm.

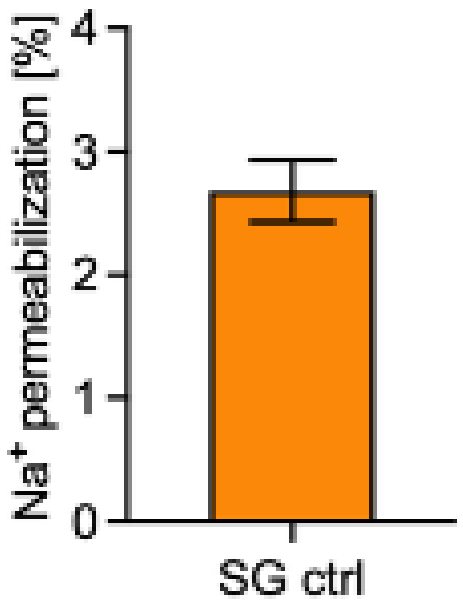

Supplementary Fig. S9. Passive Na^+ permeability control of $PMOXA_{10}$-*b*-$PDMS_{25}$ polymersomes. Sodium green (SG) fluorescence change after incubation with 100 mM NaCl, normalized to the Triton X-100 endpoint signal. Data represent the mean of three independent experiments; error bar indicates standard deviation.
